# Activities, substrate specificity, and genetic interactions of fission yeast Siw14, a cysteinyl-phosphatase-type inositol pyrophosphatase

**DOI:** 10.1128/mbio.02056-23

**Published:** 2023-09-29

**Authors:** Ana M. Sanchez, Beate Schwer, Nikolaus Jork, Henning J. Jessen, Stewart Shuman

**Affiliations:** 1 Molecular Biology Program, Sloan Kettering Institute, New York, New York, USA; 2 Gerstner Sloan Kettering Graduate School of Biomedical Sciences, New York, New York, USA; 3 Department of Microbiology and Immunology, Weill Cornell Medical College, New York, New York, USA; 4 Institute of Organic Chemistry and Centre for Integrative Biological Signaling Studies, University of Freiburg, Freiburg, Germany; 5 Spemann Graduate School of Biology and Medicine, University of Freiburg, Freiburg, Germany; Harvard Medical School, Boston, Massachusetts, USA

**Keywords:** inositol pyrophosphates, inorganic polyphosphate, pyrophosphatase, *Schizosaccharomyces pombe*, transcription termination

## Abstract

Inositol pyrophosphate 1,5-IP_8_ is a signaling molecule that regulates phosphate and polyphosphate homeostasis in the fission yeast *Schizosaccharomyces pombe*. 1,5-IP_8_ levels are dictated by a balance between the Asp1 kinase domain that converts 5-IP_7_ to 1,5-IP_8_ and two pyrophosphatases—the Asp1 pyrophosphatase domain (histidine acid phosphatase family) and the Aps1 pyrophosphatase enzyme (Nudix family)—that hydrolyze the β-phosphates of 1,5-IP_8_. Here, we characterize *S. pombe* Siw14 (SpSiw14), a cysteinyl-phosphatase family member and a homolog of *Saccharomyces cerevisiae* Siw14, as a third fission yeast pyrophosphatase implicated in inositol pyrophosphate catabolism. We find that SpSiw14’s substrate repertoire embraces inorganic pyrophosphate, inorganic polyphosphate, and the inositol pyrophosphates 5-IP_7_, 1-IP_7_, and 1,5-IP_8_, in addition to the generic substrate *p*-nitrophenylphosphate. Genetic analyses revealed that (i) elimination of the SpSiw14 protein or inactivation of the SpSiw14 pyrophosphatase by the C189S mutation had no effect on *S. pombe* growth but was lethal in the absence of Aps1 and (ii) the synthetic lethality of *siw14*∆ *aps1*∆ depended on the synthesis of 1,5-IP_8_ by the Asp1 kinase. We conclude that SpSiw14 and Aps1 pyrophosphatases have essential but redundant functions in fission yeast, and that their synthetic lethality is a consequence of the toxic effects of too much 1,5-IP_8_. Suppression of *siw14*∆ *aps1*∆ lethality by loss-of-function mutations of components of the fission yeast 3′-processing/termination machinery fortifies the case for overzealous transcription termination as the basis for 1,5-IP_8_ toxicosis.

**IMPORTANCE:**

The inositol pyrophosphate signaling molecule 1,5-IP_8_ modulates fission yeast phosphate homeostasis via its action as an agonist of RNA 3′-processing and transcription termination. Cellular 1,5-IP_8_ levels are determined by a balance between the activities of the inositol polyphosphate kinase Asp1 and several inositol pyrophosphatase enzymes. Here, we characterize *Schizosaccharomyces pombe* Siw14 (SpSiw14) as a cysteinyl-phosphatase-family pyrophosphatase enzyme capable of hydrolyzing the phosphoanhydride substrates inorganic pyrophosphate, inorganic polyphosphate, and inositol pyrophosphates 5-IP_7_, 1-IP_7_, and 1,5-IP_8_. Genetic analyses implicate SpSiw14 in 1,5-IP_8_ catabolism *in vivo*, insofar as: loss of SpSiw14 activity is lethal in the absence of the Nudix-type inositol pyrophosphatase enzyme Aps1; and *siw14*∆ *aps1*∆ lethality depends on synthesis of 1,5-IP_8_ by the Asp1 kinase. Suppression of *siw14*∆ *aps1*∆ lethality by loss-of-function mutations of 3′-processing/termination factors points to precocious transcription termination as the cause of 1,5-IP_8_ toxicosis.

## INTRODUCTION

Inositol pyrophosphates IP_7_ and IP_8_ are signaling molecules that influence eukaryotic phosphate and polyphosphate homeostasis (1). 5-IP_7_ and 1-IP_7_ differ according to whether the pyrophosphate moiety is at the 1 or 5 position of the inositol ring; 1,5-IP_8_ is pyrophosphorylated at both ring positions. 1,5-IP_8_ is synthesized from phytic acid (IP_6_) by the sequential action of inositol polyphosphate kinases Kcs1/IP6K, which converts IP_6_ to 5-IP_7_, and Asp1/Vip1/VIH/PPIP5K, which converts 5-IP_7_ to 1,5-IP_8_ (1). Asp1, Vip1, VIH, and PPIP5K—as they are named in fission yeast, budding yeast, plants, and humans, respectively—are bifunctional enzymes composed of an N-terminal kinase domain that synthesizes 1,5-IP_8_ and a C-terminal pyrophosphatase domain of the histidine acid phosphatase enzyme family that converts 1,5-IP_8_ back to 5-IP_7_ (2 – 8).

In addition to the C-terminal pyrophosphatase domain of Asp1/Vip1/VIH/PPIP5K, two other classes of pyrophosphatases are engaged in the catabolism of inositol pyrophosphates. The first class, exemplified by DIPP/Ddp1/Aps1 (from human, budding yeast, and fission yeast, respectively), are Nudix-family pyrophosphatases, defined by an ~23 aa Nudix box motif in which three glutamates comprise a binding site for catalytic magnesium ions (9 – 12). DIPP/Ddp1/Aps1 are also active in hydrolyzing inorganic polyphosphates and/or diadenosine polyphosphates (12 – 15).

The founder of the second class of pyrophosphatases is the *Saccharomyces cerevisiae* enzyme Siw14 (ScSiw14), which removes the 5-β-phosphate from 5-IP_7_ and 1,5-IP_8_ but does not hydrolyze the 1-β-phosphate of 1-IP_7_ (16, 17). ScSiw14 (281-aa) belongs to the cysteinyl-phosphatase family of phosphohydrolases, defined by a conserved active site phosphate-binding loop HCxxxxxR, that catalyzes phosphoryl transfer to water via a covalent enzyme-(cysteinyl-Sγ)-phosphate intermediate. Mutating ScSiw14’s active site, Cys214, to serine abolishes its activity. Crystal structures of N-terminally deleted versions of ScSiw14 have been solved with sulfate or citrate anions in the active site (17, 18). The plant *Arabidopsis thaliana* encodes five paralogous Siw14 homologs (named PFA-DSPs 1–5) that prefer to hydrolyze the 5-β-phosphate of inositol pyrophosphates (19). Crystal structures of N-terminally truncated plant PFA-DSP1 have been solved, either of wild-type DSP1 with its product inorganic phosphate in the active site or the inactive C150S mutant with its active site filled by its preferred substrate 5-IP_7_ (20).

In the fission yeast *Schizosaccharomyces pombe*, IP_8_ dynamics are dictated by a balance between Asp1 kinase and the pyrophosphatase enzymes that remove the 1- or 5-β-phosphate groups. *asp1*∆ cells or kinase-inactive *asp1-D333A* mutant cells are viable and contain no detectable 1,5-IP_8_ (3, 4). Pyrophosphatase-defective *asp1-H397A* cells are viable but contain elevated levels of 1,5-IP_8_ (3, 4). A key finding was that the *asp1-H397A* mutation is synthetically lethal in an *aps1*∆ strain that lacks the Nudix pyrophosphatase enzyme, signifying that too much 1,5-IP_8_ is toxic to fission yeast (21). Multiple lines of genetic, biochemical, and transcriptomic evidence cohere to show that (i) 1,5-IP_8_ acts as an agonist of precocious RNA polymerase II (Pol2) transcription termination dependent on the 3′ cleavage and polyadenylation factor (CPF) complex and (ii) 1,5-IP_8_ toxicosis results from overzealous 3′-processing/transcription termination (21 – 24).

It is not known whether or how Siw14 contributes to 1,5-IP_8_ metabolism or 1,5-IP_8_ signaling in fission yeast. The present study, in which we interrogate the biochemical activity of *S. pombe* Siw14 (SpSiw14) and its genetic interactions, is inspired by our recent observations that *siw14*∆, which has no effect *per se* on fission yeast growth, is synthetically lethal with *aps1*∆ (lacking the Nudix-type pyrophosphatase) (25). We report here that SpSiw14 hydrolyzes the generic phosphomonoester *p*-nitrophenylphosphate, the inorganic phosphoanhydride substrates pyrophosphate and polyphosphate, and the inositol pyrophosphates 5-IP_7_, 1-IP_7_, and 1,5-IP_8_. Active site mutation C189S abolishes phosphohydrolase activity *in vitro* and results in lethality with *aps1*∆ *in vivo*. Genetic suppression implicates overzealous 3′-processing/transcription termination as the basis for *siw14*∆ *aps1*∆ lethality.

## RESULTS

### Recombinant *S. pombe* Siw14 is a metal-independent phosphohydrolase

**FIG 1:**
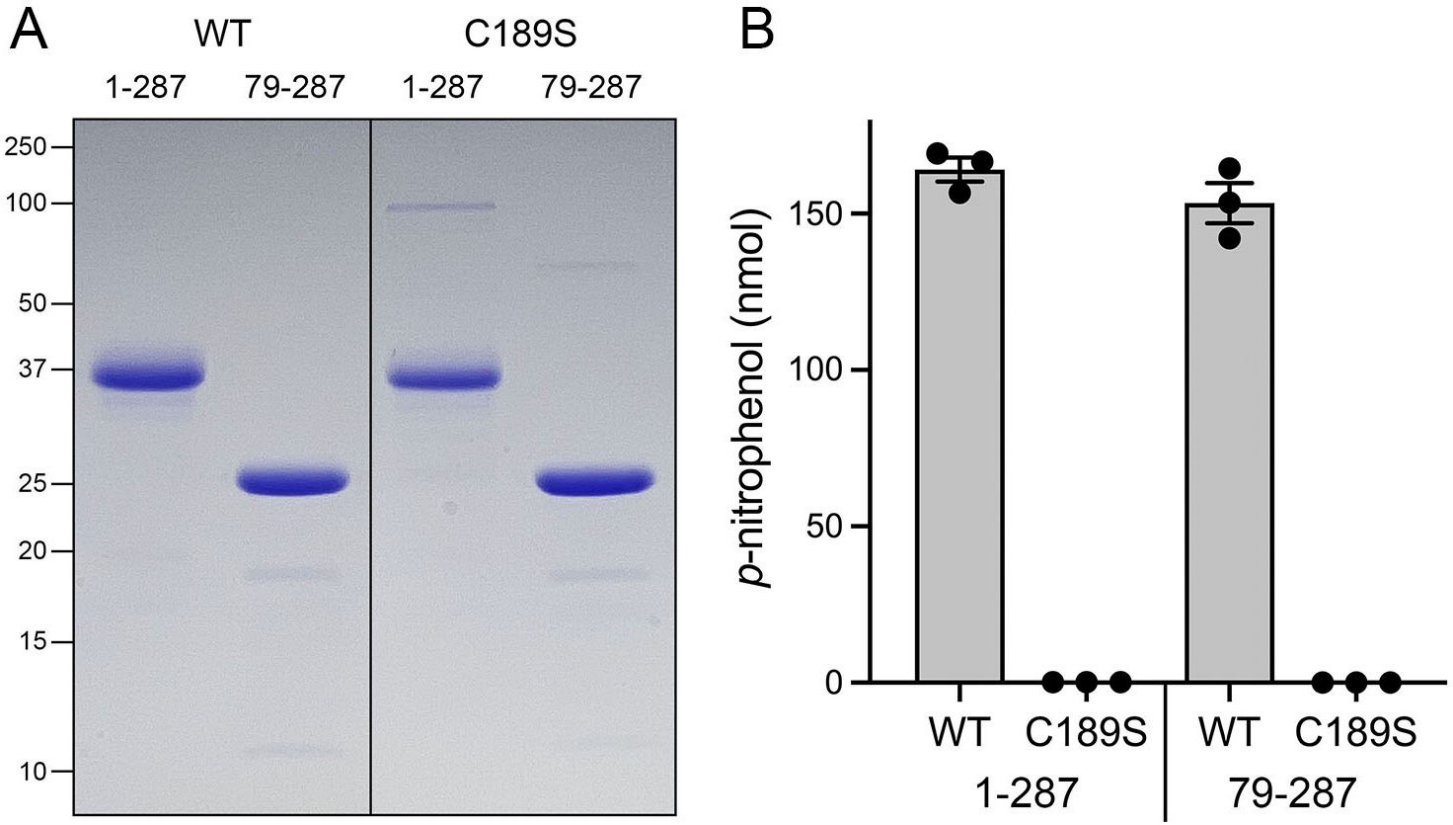
Recombinant Siw14 hydrolyzes *p*-nitrophenylphosphate. (**A**) Aliquots (5 µg) of the indicated wild-type or mutant Siw14 preparations were analyzed by SDS-PAGE. The Coomassie blue-stained gel is shown. The positions and sizes (kDa) of marker polypeptides are indicated on the left. (**B**) Phosphatase reaction mixtures (50 µL) containing 25 mM Tris-acetate, pH 5.0, 1 mM DTT, 10 mM (500 nmol) *p*-nitrophenylphosphate, and 0.8 µM (40 pmol) of the wild-type or mutant Siw14 preparations were incubated at 37°C for 60 min. The extent of *p*-nitrophenol production is plotted. Data in the bar graph are the average of three independent assays ± SEM.

We produced recombinant full-length (aa 1–287) wild-type SpSiw14 and a full-length active site mutant C189S in *Escherichia coli* as His_10_Smt3 fusions and isolated them from soluble bacterial extracts by Ni-affinity chromatography. The tags were removed by treatment with the Smt3 protease Ulp1, and the SpSiw14 proteins were recovered free of His_10_Smt3 after a second round of Ni-affinity chromatography. In parallel, we produced and purified N-terminally truncated versions: wild-type SpSiw14-(79-287) and SpSiw14-(79-287)-C189S. The proteins were subjected to a final gel filtration step, during which they eluted as single peaks consistent with monomeric native size. SDS-PAGE revealed comparable purity of the recombinant wild-type and C189S proteins of the expected sizes (Fig. 1A).

To interrogate SpSiw14 enzymatic function, we tested activity with the generic phosphomonoesterase substrate *p*-nitrophenylphosphate. Hydrolysis of *p*-nitrophenylphosphate liberates *p*-nitrophenol, which is quantified by its absorbance at 410 nm. We found that full-length SpSiw14 and truncated SpSiw14-(79-287) readily converted 10 mM *p*-nitrophenylphosphate into *p*-nitrophenol during a 60-min reaction at 37°C in the absence of a divalent cation. Equivalent amounts of SpSiw14-C189S and SpSiw14-(79-287)-C189S were catalytically inert (Fig. 1B).

**FIG 2:**
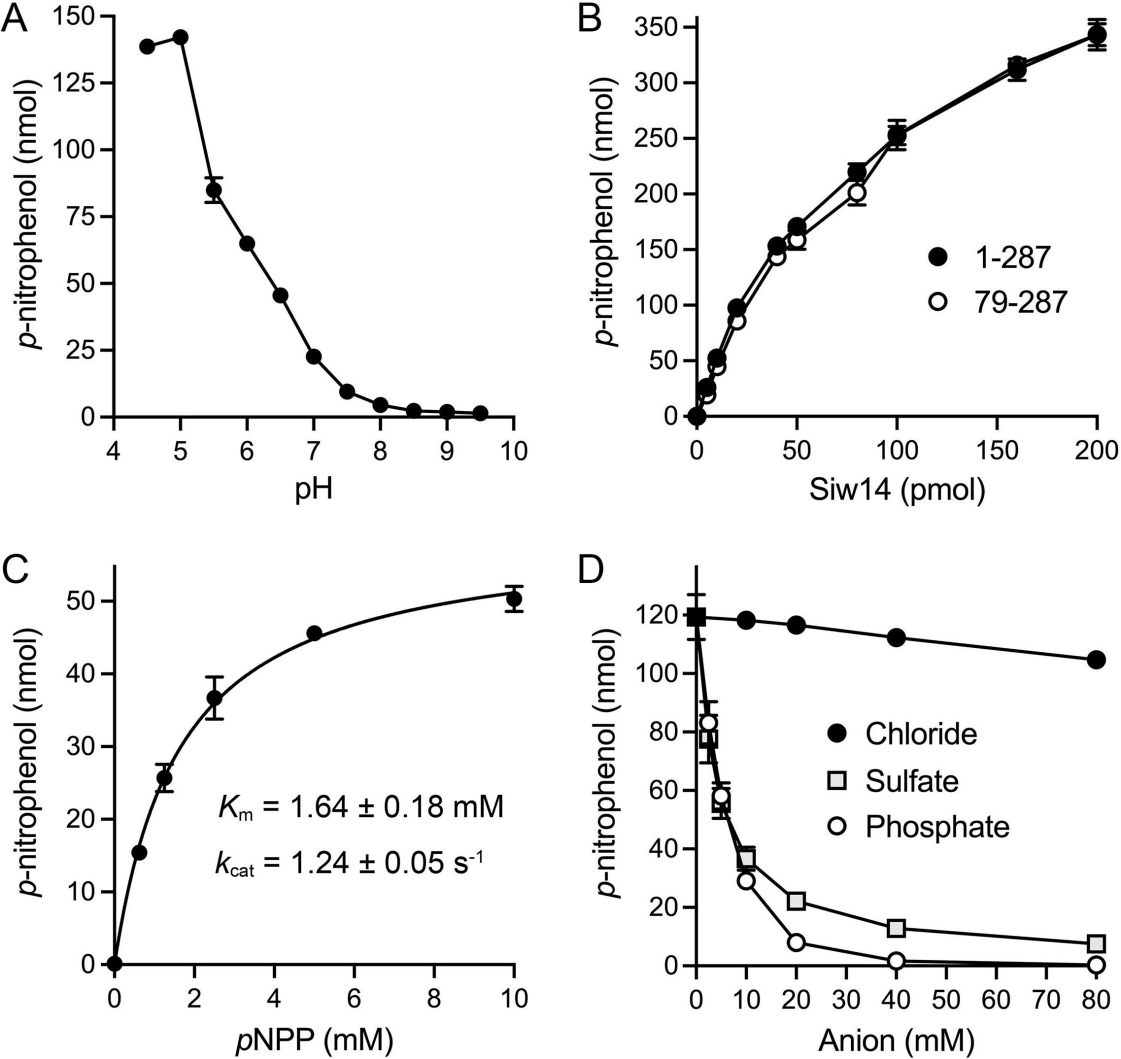
Characterization of the Siw14 *p*-nitrophenylphosphatase activity. (**A**) pH profile. Reaction mixtures (50 µL) containing 25 mM buffer (either Tris-acetate pH 4.5, 5.0, 5.5, 6.0, 6.5 or Tris-HCl pH 7.0, 7.5, 8.0, 8.5, 9.0, 9.5), 1 mM DTT, 10 mM (500 nmol) *p*-nitrophenylphosphate, and 0.8 µM (40 pmol) of Siw14-(1-287) were incubated at 37°C for 60 min. The extent of *p*-nitrophenol production is plotted. Each datum is the average of two independent experiments ± range. (**B**) Siw14-(1-287) and Siw14-(79-287) titration. Reaction mixtures (50 µL) containing 25 mM Tris-acetate, pH 5.0, 1 mM DTT, 10 mM (500 nmol) *p*-nitrophenylphosphate, and increasing amounts of Siw14 as specified on the x-axis were incubated at 37°C for 60 min. *p*-Nitrophenol production is plotted as a function of input Siw14. (**C**) Steady-state kinetic parameters. Reaction mixtures (50 µL) containing 25 mM Tris-acetate, pH 5.0, 1 mM DTT, 0.8 µM (40 pmol) Siw14-(1-287), and increasing concentrations of *p*-nitrophenylphosphate as specified on the x-axis were incubated at 37°C for 20 min. *p*-Nitrophenol production is plotted as a function of substrate concentration, and the data were fit to the Michaelis-Menten equation in Prism. (**D**) Inhibition by phosphate. Reaction mixtures (50 µL) containing 25 mM Tris-acetate, pH 5.0, 1 mM DTT, 10 mM (500 nmol) *p*-nitrophenylphosphate, and 0.7 µM (35 pmol) Siw14-(1-287) were supplemented with sodium chloride, sodium sulfate, or sodium phosphate at the concentrations specified on the x-axis. The reaction mixtures were incubated at 37°C for 60 min. The extent of *p*-nitrophenol production is plotted as a function of added anion concentration. The data plotted in panels B–D are the averages of three independent experiments ± SEM.

SpSiw14 *p*-nitrophenylphosphatase was optimal at pH 4.5 to 5.0; activity declined steadily as the pH was increased to 8.0 (Fig. 2A). The extent of *p*-nitrophenylphosphate hydrolysis during a 60-min reaction increased linearly with input full-length SpSiw14 or truncated SpSiw14-(79-287) up to 25 pmol and continued to increase with a shallower slope in the range of 25 to 200 pmol, at which point 70% of the substrate was consumed (Fig. 2B). From the slopes of the SpSiw14 and SpSiw14-(79-287) titration curves in the initial linear phase, we calculated specific activities of 4.9 and 4.3 nmol of *p*-nitrophenol formed per pmol of SpSiw14 and SpSiw14-(79-287), respectively. SpSiw14 activity during a 20-min reaction under steady-state conditions (<10% of substrate consumed by 0.8 µM enzyme) displayed a hyperbolic dependence on *p*-nitrophenylphosphate concentration (Fig. 2C). Fitting the data to the Michaelis-Menten equation yielded *K*_*m*_ and *k*_cat_ values of 1.64 ± 0.18 mM and 74.4 ± 2.7 min^−1^, respectively. All ensuing experiments were performed with full-length SpSiw14 proteins.

### SpSiw14 activity is inhibited by phosphate and sulfate

We supplemented the standard reaction mixtures containing 10 mM *p*-nitrophenylphosphate with increasing concentrations of sodium phosphate (2.5, 5, 10, 20, 40, and 80 mM). Parallel reactions were supplemented with the equivalent concentrations of sodium chloride or sodium sulfate. Phosphate and sulfate elicited a similar concentration-dependent inhibition of SpSiw14 phosphatase activity with an IC_50_ of ~5 mM each (Fig. 2D). These results suggest that inorganic phosphate (a reaction product) and sulfate (a mimetic of phosphate) bind to the active site as well as, or better than, the 10 mM *p*-nitrophenylphosphate substrate. Phosphatase activity was inhibited completely at 40 to 80 mM phosphate; at 80 mM sulfate, the residual activity was 6% of the unsupplemented control. By contrast, chloride had relatively little impact, even up to 80 mM, at which point activity was diminished by only 12% compared to the unsupplemented control (Fig. 2D). We also tested whether magnesium affected Siw14 activity by supplementing reaction mixtures with 5, 10, or 20 mM MgCl_2_. The extent of *p*-nitrophenylphosphate hydrolysis in 5, 10, and 20 mM MgCl_2_ was 94%, 91%, and 80%, respectively, of the unsupplemented control (not shown).

### SpSiw14 hydrolyzes inorganic pyrophosphate and polyphosphate

**FIG 3:**
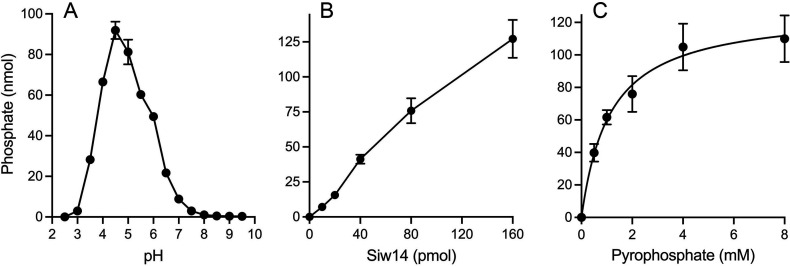
Siw14 hydrolyzes inorganic pyrophosphate. (**A**) pH profile. Reaction mixtures (100 µL) containing 25 mM buffer (either citric acid pH 2.5, 3.0, 3.5, 4.0; Tris-acetate pH 4.5, 5.0, 5.5, 6.0, 6.5; or Tris-HCl pH 7.0, 7.5, 8.0, 8.5, 9.0, 9.5), 1 mM DTT, 2 mM (200 nmol) sodium pyrophosphate, and 0.8 µM (80 pmol) Siw14-(1-287) were incubated at 37°C for 30 min. The extent of phosphate production is plotted as a function of pH. Each datum is the average of two separate titration experiments ± range. (**B**) Siw14 titration. Reaction mixtures (100 µL) containing 25 mM Tris-acetate, pH 5.0, 1 mM DTT, 2 mM (200 nmol) sodium pyrophosphate, and increasing amounts of Siw14-(1-287) as specified on the x-axis were incubated at 37°C for 30 min. The extent of phosphate production is plotted. (**C**) Steady-state kinetic parameters. Reaction mixtures (100 µL) containing 25 mM Tris-acetate, pH 5.0, 1 mM DTT, 0.8 µM (80 pmol) Siw14-(1-287), and increasing concentrations of sodium pyrophosphate as specified on the x-axis were incubated at 37°C for 30 min. Phosphate production is plotted as a function of pyrophosphate concentration, and the data were fit to the Michaelis-Menten equation in Prism. The data plotted in panels B and C are the averages of three independent experiments ± SEM.

SpSiw14 was reacted for 30 min with 2 mM inorganic pyrophosphate (PP_i_), and the formation of inorganic phosphate (P_i_) products was determined via colorimetric assay using the Malachite Green reagent. Hydrolysis of PP_i_ displayed a bell-shaped dependence on the pH of the reaction mixture. Pyrophosphatase activity was optimal at pH 4.5 to 5.0; activity declined steadily as the pH was decreased to ≤3.0 or increased to ≥8.0 (Fig. 3A). The extent of PP_i_ hydrolysis was proportional to input SpSiw14 (Fig. 3B). From the slope of the titration curve in the initial linear phase, we calculated a specific activity of 953 pmol of P_i_ formed (i.e., 477 pmol of PP_i_ hydrolyzed) per pmol of SpSiw14. Pyrophosphatase activity displayed a hyperbolic dependence on PP_i_ concentration (Fig. 3C). Fitting the data to the Michaelis-Menten equation yielded a *K*_*m*_ value of 1.13 ± 0.34 mM PP_i_ and an apparent *k*_cat_ of 26.6 ± 4.9 min^−1^ with respect to PP_i_ consumed per enzyme.

**FIG 4:**
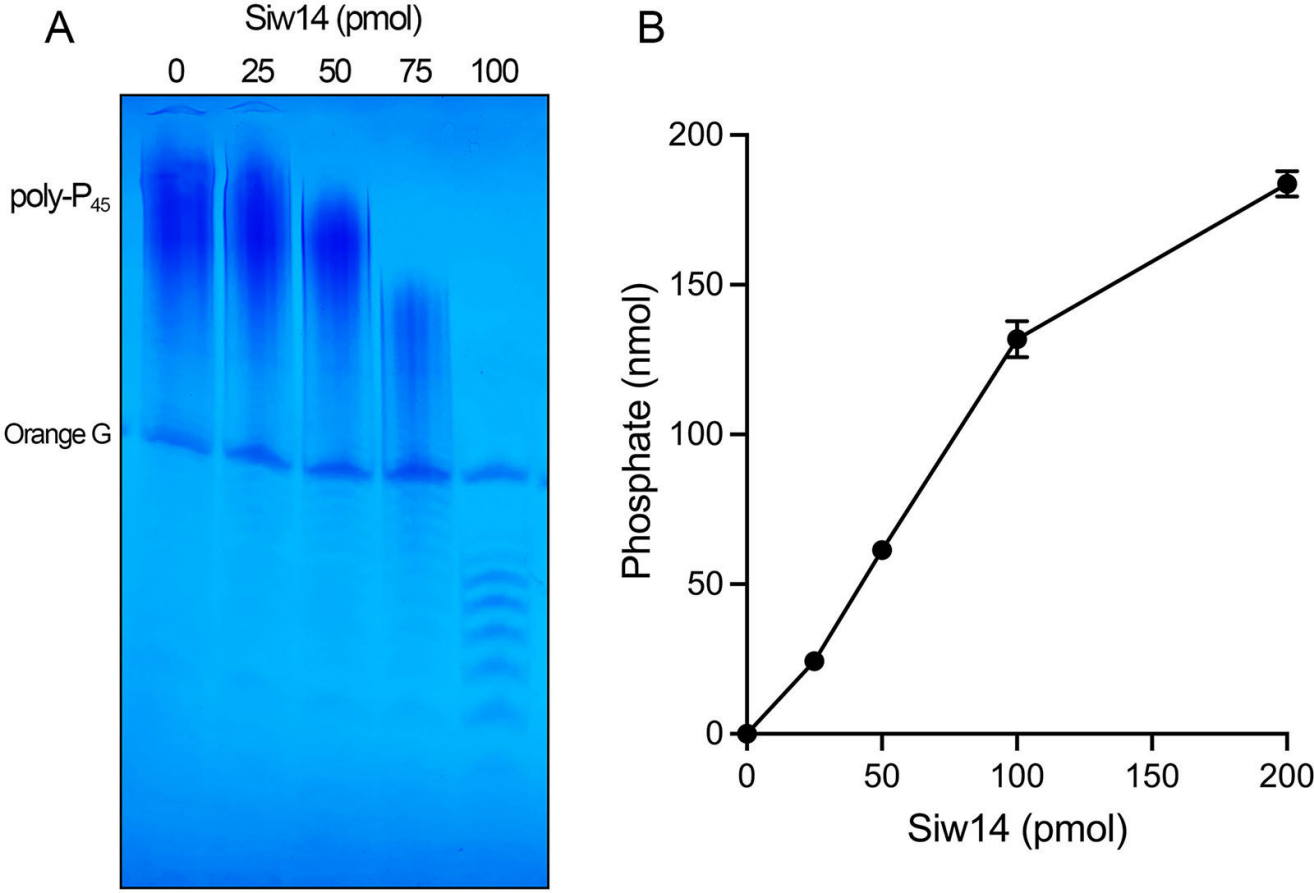
Siw14 hydrolyzes inorganic polyphosphate. (**A**) Reaction mixtures (10 µL) containing 50 mM Tris-acetate, pH 5.0, 0.2 mM poly-P_45_, and 0, 25, 50, 75, or 100 pmol of Siw14-(1-287) were incubated for 30 min at 37°C. Reaction products were resolved by PAGE and visualized by toluidine blue staining. (**B**) Reaction mixtures (20 µL) containing 50 mM Tris-acetate, pH 5.0, 0.2 mM poly-P_45_ (200 nmol total orthophosphate), and increasing concentrations of Siw14(1-287) were incubated for 30 min at 37°C. The extent of orthophosphate production is plotted. The data are the averages of three independent experiments ± SEM.

We then reacted SpSiw14 with inorganic polyphosphate with an average linear polymer chain length of 45 (poly-P_45_). The products were analyzed by electrophoresis through a 36% polyacrylamide gel, and the polyphosphate chains were visualized by staining the gel with toluidine blue (Fig. 4A). Increasing input SpSiw14 effected a progressive shortening of the polyphosphate chains. Deploying the malachite green assay, we found that the reaction of SpSiw14 with poly-P_45_ generated inorganic phosphate as a reaction product (Fig. 4B). The extent of P_i_ release during a 30-min reaction increased with input SpSiw14. At 200 pmol SpSiw14, 92% of the input poly-P was converted to free phosphate. From the slope of the titration curve in the linear range, we calculated that 1.34 nmol of phosphate was released per pmol of SpSiw14, which translates into a turnover number of 45 min^−1^. We conclude that SpSiw14 has vigorous exopolyphosphatase activity.

Poly-P is generated *in vivo* by a heterotrimeric membrane-associated VTC complex (comprising Vtc4, Vtc2, and Vtc1 subunits) that synthesizes poly-P and simultaneously imports the poly-P into the yeast vacuole (26). Poly-P levels and polymer chain length are determined by a dynamic balance between synthesis by the VTC poly-P polymerase and catabolism by exopolyphosphatase or endopolyphosphatase enzymes. *S. pombe* encodes three annotated polyphosphatases: Ppx1, SPBC713.07c, and SPCC1840.07c, of which the latter two are designated as vacuolar. To query whether SpSiw14 contributes significantly to inorganic polyphosphate homeostasis *in vivo*, we monitored the total polyphosphate pool of wild-type and *siw14*∆ cells (three biological replicates each) via polyacrylamide gel electrophoresis and staining with toluidine blue (Fig. S1). The abundance and length of the linear polyphosphate species were apparently the same in both strains. The lack of impact of *siw14*∆ on poly-P *in vivo* could simply reflect differential localization, i.e., the cellular poly-P pool is predominantly vacuolar, whereas Siw14 is annotated in Pombase as localizing to the nucleus and cytoplasm.

### SpSiw14 hydrolyzes inositol pyrophosphates

**FIG 5:**
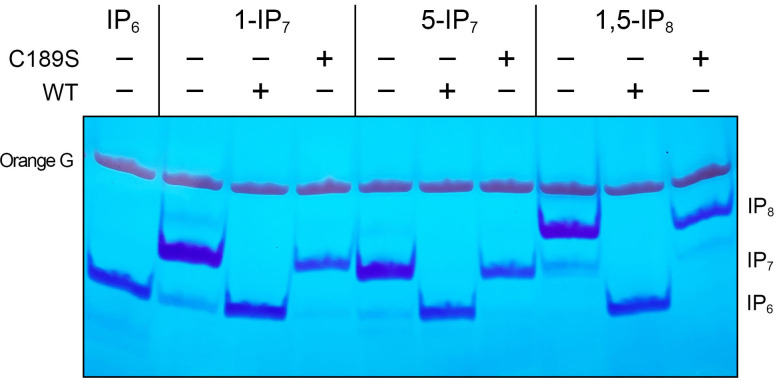
Siw14 is an inositol pyrophosphate pyrophosphatase. Reaction mixtures (20 µL) containing 30 mM Tris-acetate, pH 5.0, 50 mM NaCl, 0.25 mM (5 nmol) inositol pyrophosphate (1-IP_7_, 5-IP_7_, or 1,5-IP_8_), and 3.9 µM (78 pmol) wild-type or C189S Siw14-(1-287) (where indicated by +) were incubated for 60 min at 37°C. The reaction products were resolved by PAGE and visualized by toluidine blue staining.

**FIG 6:**
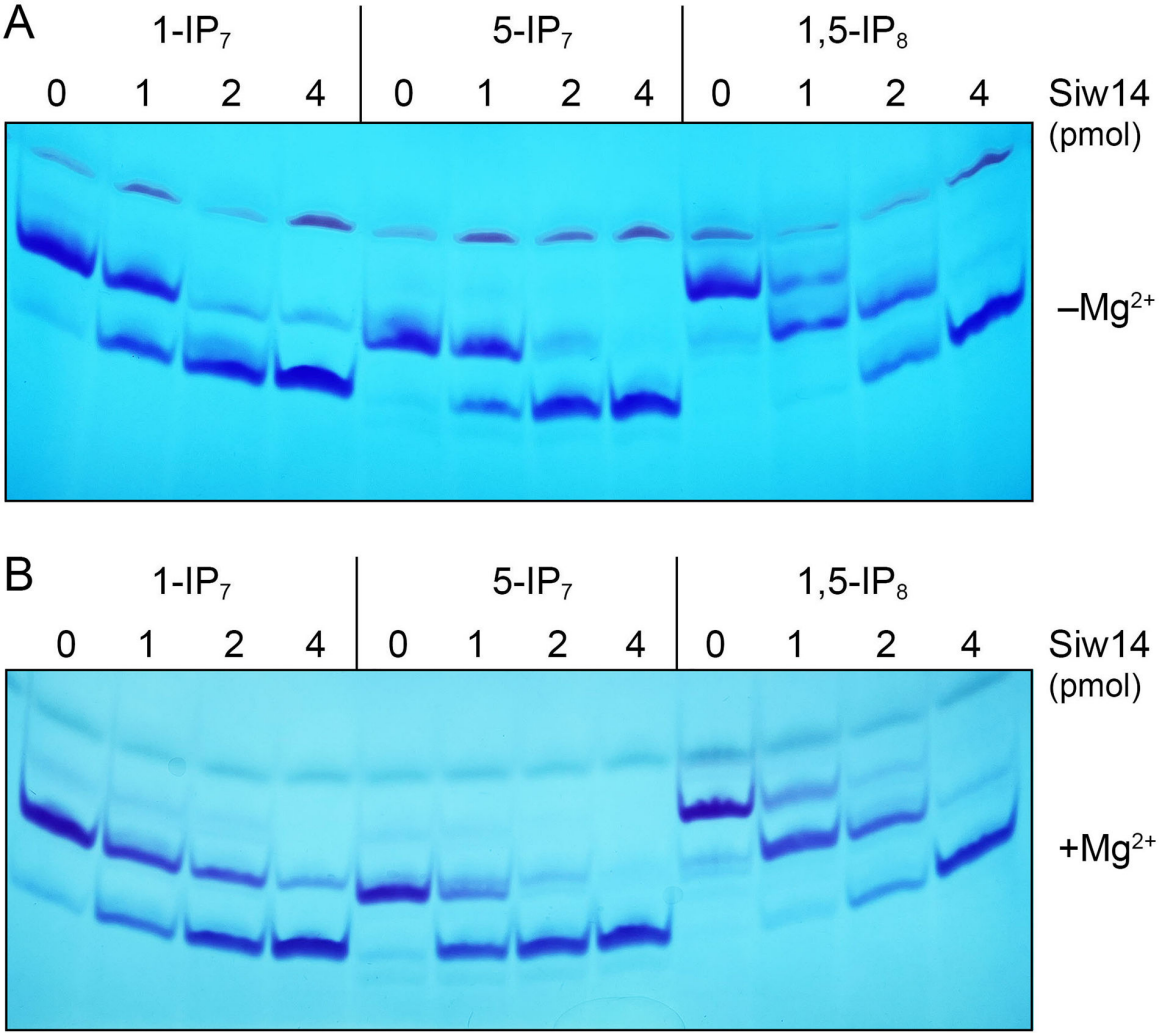
Siw14 hydrolyzes the 1-β and 5-β phosphate groups with similar efficiency. Reaction mixtures (10 µL) containing 30 mM Tris-acetate, pH 5.0, 25 mM NaCl, 0.5 mM (5 nmol) inositol pyrophosphate (1-IP_7_, 5-IP_7_, or 1,5-IP_8_), in the absence (panel A) or presence of 1 mM MgCl_2_ (panel B), and increasing concentrations of wild-type Siw14 as specified were incubated for 30 min at 37°C. The reaction products were resolved by PAGE and visualized by toluidine blue staining.

SpSiw14 or SpSiw14-C189S (3.9 µM, 78 pmol) was reacted for 60 min with 0.25 mM (5 nmol) 1-IP_7_, 5-IP_7_, or 1,5-IP_8_, and the products were analyzed by electrophoresis through a 36% polyacrylamide gel. The polyphosphorylated species were visualized by staining the gel with toluidine blue (Fig. 5). Wild-type SpSiw14 effected quantitative conversion of each inositol pyrophosphate substrate to IP_6_. By contrast, SpSiw14-C189S was inert in the hydrolysis of inositol pyrophosphates (Fig. 5). SpSiw14 back-titration (1, 2, and 4 pmol) revealed that 1-IP_7_ and 5-IP_7_ were hydrolyzed to IP_6_ with similar efficiency, and that 1,5-IP_8_ was converted to an IP_7_ species prior to the formation of the IP_6_ end-product (Fig. 6A). Based on the observations that 2 pmol of SpSiw14 sufficed to convert virtually all the input IP_8_ (5 nmol) to a mixture of IP_7_ and IP_6_, we estimated a turnover number of 83 min^−1^ for the SpSiw14 inositol pyrophosphatase, which agrees with the turnover number for hydrolysis of *p*-nitrophenylphosphate.

Our findings here that fission yeast SpSiw14 does not display a marked preference for 5-IP_7_ versus 1-IP_7_ contrast with the properties reported for *S. cerevisiae* Siw14 (16, 17). In the case of the plant Siw14 homologs, it was found that (i) PFA-DSP1, -DSP2, and -DSP4 hydrolyzed either 5-IP_7_ or 1-IP_7_ (at 0.33 mM concentration) when the reactions were performed in the absence of a divalent cation and (ii) inclusion of 1 mM magnesium in the reaction mixtures conferred selectivity for 5-IP_7_ (19). The selectivity of PFA-DSP1 in the presence of magnesium appears to result from magnesium inhibiting its action on 1-IP_7_ but not 5-IP_7_ (19). We find that when the enzyme titration experiments were performed in the presence of 1 mM magnesium, SpSiw14 was approximately twofold more active at hydrolyzing 5-IP_7_ versus 1-IP_7_ (Fig. 6B). Magnesium had no apparent effect on SpSiw14 pyrophosphatase activity with 1,5-IP_8_ (Fig. 6A and B).

### *siw14-C189S* is synthetically lethal with *aps1∆*

**FIG 7:**
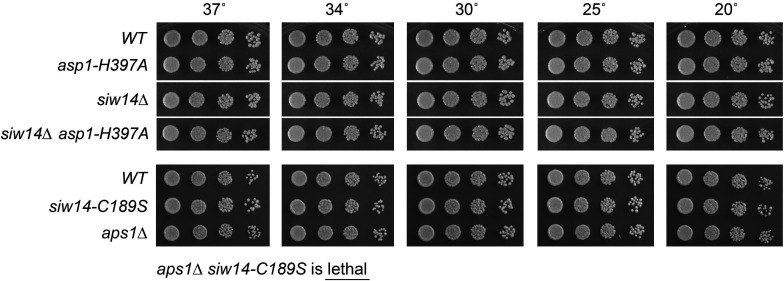
*siw14-C189S* is synthetically lethal with *aps1*∆. *S. pombe* strains with the indicated *asp1*, *siw14*, and *aps1* alleles were inoculated in YES broth and grown at 30°C. Exponentially growing cultures were adjusted to an *A*_600_ of 0.1, and aliquots (3 µL) of serial fivefold dilutions were spotted on YES agar and then incubated at the temperatures specified. *aps1*∆ was synthetically lethal with *siw14-C189S*.

None of the three known fission yeast inositol pyrophosphate pyrophosphatase activities is essential *per se* for vegetative growth, i.e., the pyrophosphatase-defective *asp1-H397A* strain and the *aps1*∆ and *siw14*∆ null strains grow well on yeast extract with supplements (YES) agar at 20°C to 37°C (Fig. 7). However, *asp1-H397A* is synthetically lethal with *aps1*∆ (21), suggesting that simultaneous ablation of these two pyrophosphatases results in the accumulation of toxic levels of 1,5-IP_8_. When attempting to construct a *siw14*∆ *aps1*∆ double mutant strain via mating and sporulation, we found that the *siw14*∆ and *aps1*∆ alleles were synthetically lethal. To wit: (i) we were unable to obtain viable double mutants after screening a large population of haploid progeny of the genetic cross; and (ii) wild-type progeny and the differentially marked *siw14*∆ (on chromosome II) and *aps1*∆ (on chromosome I) single mutants were recovered at the expected frequencies (25). This result signified that the SpSiw14 and the Aps1 pyrophosphatases have essential but redundant functions in fission yeast. By contrast, we readily isolated a *siw14*∆ *asp1-H397A* double mutant that grew as well as wild type on YES agar at all temperatures (Fig. 7).

To see if SpSiw14 pyrophosphatase activity is pertinent to the synthetic lethality of *siw14*∆ with *aps1*∆, we crossed the marked *siw14-C189S* and *aps1*∆ strains and screened for double-mutants by random spore analysis. The *siw14-C189S* active site mutant phenocopied *siw14*∆ with respect to synthetic lethality with *aps1*∆ (Fig. 7), consistent with the idea that inactivation of these two pyrophosphatases at once results in the accumulation of toxic levels of 1,5-IP_8_ and/or another phosphoanhydride-containing metabolite.

### The lethality of *siw14*∆ *aps1*∆ depends on Asp1 kinase activity

**FIG 8:**
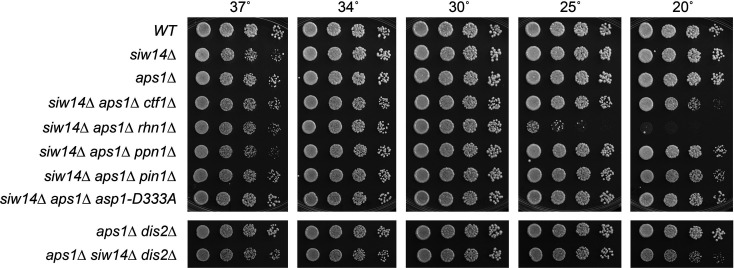
Lethality of *siw14*∆ *aps1*∆ depends on Asp1 kinase activity, CPF subunits Ctf1, Dis2, and Ppn1, prolyl isomerase Pin1, and termination factor Rhn1. Fission yeast strains with the *siw14*∆ allele in various combinations with *aps1*∆ and CPF subunit mutations or the Asp1 kinase-inactivating mutation D333A were spot tested for growth at the temperatures specified.

If the synthetic lethality of *siw14*∆ *aps1*∆ is due to 1,5-IP_8_ toxicity, then the lethality should be suppressed by the kinase-dead *asp1-D333A* allele (6, 8). Indeed, after crossing *aps1*∆ *asp1-D333A* and *siw14*∆ strains and random spore analysis, we obtained viable *siw14*∆ *aps1*∆ *asp1-D333A* cells that grew as well as wild type on YES agar (Fig. 8).

### The lethality of *siw14*∆ *aps1*∆ depends on CPF subunits, Rhn1, Pin1, and the Pol2 carboxy-terminal domain threonine-4

The fission yeast CPF is a 13-subunit protein assembly responsible for the 3′ processing of nascent Pol2 transcripts that precedes and abets Pol2 transcription termination (27). Five of the CPF subunits (Ctf1, Ssu72, Dis2, Ppn1, and Swd22) are dispensable for growth. Dis2 and Ssu72 are phosphoprotein phosphatase enzymes. Rhn1 is an inessential Pol2 termination factor that recognizes the Thr4-PO_4_ mark on the carboxy-terminal domain (CTD) of the Rpb1 subunit of Pol2 (28). Pin1 is a peptidyl prolyl isomerase that abets the function of Ssu72, a *cis*-proline-dependent CTD phosphatase (29).

**FIG 9:**
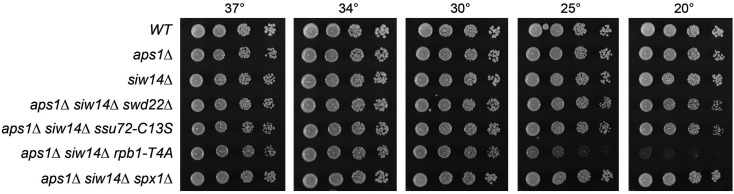
Lethality of *siw14*∆ *aps1*∆ depends on CPF subunits Swd22 and Ssu72, CTD threonine-4, and Spx1. *siw14*∆, *aps1*∆, and the indicated triple-mutant progeny of genetic crosses were spot-tested for growth on YES agar at the temperatures specified.

A key question is whether the lethality of the *siw14*∆ *aps1*∆ strain arises from unconstrained precocious transcription termination caused by too much 1,5-IP_8_. If so, then it might be expected that the lethality would be ameliorated by mutations in the 3′-processing/termination machinery. To test this idea, we crossed our series of *aps1*∆ *CPF/pin1/rhn1* double-mutants to *siw14*∆ then sporulated the resulting diploids and screened random spores for each of the differentially marked loci of interest. In this way, we recovered viable *siw14*∆ *aps1*∆ *ctf1*∆, *siw14*∆ *aps1*∆ *swd22*∆, *siw14*∆ *aps1*∆ *ppn1*∆, *siw14 aps1*∆ *pin1*∆, *siw14*∆ *aps1*∆ *dis2*∆, and *siw14*∆ *aps1*∆ *ssu72-C13S* haploid strains that grew like wild type on YES agar at 25°C, 30°C, and 34°C and, in some cases, grew slowly at 37°C or 20°C, as gauged by colony size (Fig. 8 and 9). The viable *siw14*∆ *aps1*∆ *rhn1*∆ triple mutant grew well at 30°C–37°C but displayed a severe cold-sensitive growth defect at 20°C and 25°C (Fig. 8). These results suggest that the synthetic lethality of *siw14*∆ *aps1*∆ is a consequence of IP_8_-driven precocious termination that depends on CPF, Pin1, and Rhn1.

We tested if *rpb1-CTD-T4A*, which negatively affects 3′-processing/termination (22, 30), would also suppress the lethality of *siw14*∆ *aps1*∆. After crossing *aps1*∆ *CTD-T4A* and *siw14*∆ strains and random spore analysis, we obtained viable triple mutant *siw14*∆ *aps1*∆ *CTD-T4A* cells that grew on YES agar at 30°C to 37°C but were severely sick at 25°C and 20°C (Fig. 9).

### *siw14*∆ *aps1*∆ lethality depends on Spx1

Deleting the inositol pyrophosphate-binding RING-domain ubiquitin ligase Spx1 (or alanine mutations of its inositol pyrophosphate-binding site or zinc-binding cysteines) suppresses the 1,5-IP_8_ toxicosis of *asp1* alleles *STF6* and *STF9* (24). Spx1 is proposed to be a mediator of the inositol pyrophosphate signal that leads to precocious transcription termination. Here, we found by crossing *aps1*∆ *spx1*∆ and *siw14*∆ that *spx1*∆ suppressed the synthetic lethality of *siw14*∆ *aps1*∆, such that the *siw14*∆ *aps1*∆ *spx1*∆ triple mutant grew as well as wild type on YES agar at all temperatures (Fig. 9).

### Transcriptome profiling of *siw14*∆ cells and comparison to *aps1*∆

We performed RNA-seq on poly(A)^+^ RNA isolated from *siw14*∆ cells and from the parental wild-type strain. cDNAs obtained from three biological replicates (using RNA from cells grown to mid-log phase in YES medium at 30°C) were sequenced for each strain. In the data sets, 86%–88% of the sequence reads were mapped to unique genomic loci (Table S1). Read densities (RPKM) for individual genes were highly reproducible between biological replicates (Pearson coefficients of 0.968 to 0.984) (Table S2). A cutoff of ±2-fold change in normalized transcript read level and an adjusted *P*-value of ≤0.05 were the criteria applied to derive an initial list of differentially expressed annotated loci in the *siw14*∆ mutant versus the wild-type control. We then focused on differentially expressed genes with average normalized read counts ≥100 in either the *siw14*∆ or wild-type strains to eliminate polyadenylated transcripts that were expressed at very low levels in vegetative cells. We thereby identified only 2 annotated protein-coding RNAs that were upregulated and 10 protein-coding RNAs (other than the *siw14* mRNA) that were downregulated in *siw14*∆ cells (Table S3). We surmise that Siw14 has little impact on gene expression in an otherwise wild-type cell.

Our prior RNA-seq analysis of *aps1*∆ cells highlighted 19 coding genes that were upregulated, including *pho1*, *pho84*, and *tgp1* that comprise the *PHO* regulon (21). Expression of the *PHO* genes in phosphate-replete cells is repressed by upstream lncRNA-mediated transcription interference. Derepression of *pho1* mRNA and Pho1 acid phosphatase enzyme activity is a sensitive indicator of genetic mutations that elicit precocious termination of upstream *prt* lncRNA transcription. The present finding by RNA-seq that the *PHO* genes were unaffected in *siw14*∆ cells resonates with our previous observation that *siw14*∆ had no effect on the level of cell-surface Pho1 acid phosphatase activity (25). In *aps1*∆ cells, 28 genes were downregulated, including 4 genes of the iron homeostasis regulon (*frp1*, *fip1*, *fio1*, and *srx1*). The noteworthy overlap between the *siw14*∆ and *aps1*∆ transcriptome data sets was that 6 of the 10 genes downregulated in *siw14*∆ were also downregulated in *aps1*∆: these being *frp1* (ferric-chelate reductase), *mei2* (RNA binding protein), *SPAC15E1.02c* (DUF1761 family protein), *SPAC637.03* (DUF1774 family protein), *SPCC70.08c* (methyltransferase), and *SPAC26H5.09c* (oxidoreductase involved in NADPH regeneration) (Table S3).

## DISCUSSION

The results herein illuminate the biochemical activities and genetic interactions of the fission yeast pyrophosphatase SpSiw14. The 287-aa SpSiw14 protein is predicted to consist of a disordered N-terminal 80-aa segment fused to a cysteinyl-phosphatase enzyme fold (alphafold.ebi.ac.uk/entry/Q9UUF3). Our initial purification and characterization of recombinant SpSiw14 proteins established its activity as a metal-independent *p*-nitrophenylphosphatase that is strictly dependent on the active site Cys189 thiol but unaffected by deletion of the disordered N-terminus, which has no primary structure similarity to the dispensable N-terminal segment of *S. cerevisiae* Siw14 (17) or the N-terminus of *Arabidopsis* PFA-DSP1. The catalytic domain of SpSiw14 is, by alignment of its primary structure (Fig. S2) and its predicted tertiary structure, highly similar to the crystal structures of ScSiw14 and plant PFA-DSP1 (17, 18, 20). SpSiw14 has a C-terminal peptide extension not found in the other two enzymes, of which the last 18-aa are predicted to be disordered (Fig. S2). The ensuing biochemical analysis of SpSiw14 reported here was performed with the full-length enzyme.

The salient findings were that SpSiw14 has a broader spectrum of pyrophosphatase activities than had been reported in previous studies of yeast and plant enzymes, whereby SpSiw14’s substrate repertoire embraces inorganic pyrophosphate, inorganic polyphosphate, and the inositol pyrophosphates 5-IP_7_, 1-IP_7_, and 1,5-IP_8_, in addition to the generic substrate *p*-nitrophenylphosphate. The turnover numbers for these phospho-substrates, derived from steady-state kinetics or estimated from enzyme-specific activity, were similar: 74 min^−1^ for *p*-nitrophenylphosphate; 27 min^−1^ for inorganic pyrophosphate; 45 min^−1^ for inorganic polyphosphate; and 83 min^−1^ for 1,5-IP_8_. The reaction rates of SpSiw14 are in the same range as the *k*_cat_ values reported for ScSiw14: 79 min^−1^ for 5-IP_7_ and 38 min^−1^ for IP_8_ (17).

SpSiw14 activity displays a bell-shaped pH profile with optimal activity at pH 4.5 to 5.0, a steep fall-off at lower pH, and a somewhat more gradual decline at higher pH. This profile suggests the involvement of at least two essential pH-sensitive moieties in the catalytic mechanism, which need to be deprotonated and protonated, respectively. One of these is the active site cysteine-189 that, as a deprotonated thiolate, attacks the terminal phosphate of the substrate to form an enzyme—(cysteinyl-Sγ)-phosphate intermediate. Studies of *Yersinia* PTPase (a founder of the cysteinyl-phosphatase family) documented an acidic pH optimum (pH 5.0) for *p*-nitrophenylphosphate hydrolysis and a p*K*_a_ of 4.67 for the active site cysteine thiol (31, 32), which is significantly lower than the expected cysteine p*K*_a_ of 8.5. Ensuing crystal structures of many cysteinyl-phosphatase family members led to the insight that the cysteine thiolate is stabilized by a surrounding network of hydrogen bond donors provided by a threonine side chain and several main-chain amides of the active site phosphate-binding loop H **C** xxxxxR **T**. As to the nature of the Siw14 moiety that needs to be protonated, we can speculate that this might be a conserved histidine in the Siw14-specific phosphate-binding loop HCxRGK **H** RT (Fig. S2). This histidine engages in two hydrogen bonds between Nδ and Nε in the ligand-bound structures of the plant Siw14-type phosphatase (20). However, deprotonation of this histidine might not account for the virtually total loss of SpSiw14 phosphatase activity at alkaline pH, insofar as an alanine mutation of the corresponding histidine in ScSiw14 elicited only a fivefold decrement in pyrophosphatase activity with 5-IP_7_ (17). Changing this histidine to aspartate in the plant enzyme resulted in a 20-fold decrease in pyrophosphatase activity with 5-IP_7_ (20).

SpSiw14 is sensitive to product inhibition by inorganic phosphate in the low millimolar range and is similarly sensitive to inhibition by sulfate. The initially reported *p*-nitrophenylphosphatase activity of recombinant *S. cerevisiae* Siw14, purified as a GST-tagged fusion protein, was notable for its very low apparent *k*_cat_ of 4.4 × 10^−7^ s^−1^ (16), which is slower than the presently reported *k*_cat_ of 1.24 s^−1^ for SpSiw14. While the low turnover was potentially attributed to a high fraction of catalytically inactive protein in the preparation, the enzyme assays in the initial study were conducted in the presence of 10 mM magnesium sulfate (16) and thus likely prone to sulfate inhibition. Changing the expression vector and purification procedures—and, coincidentally, the elimination of sulfate from the enzyme reaction mixtures—resulted in a much more active recombinant *S. cerevisiae* Siw14 preparation that was used by Wang and colleagues for structural and functional studies (17).

Inhibition of Siw14 by inorganic phosphate has potential relevance for fission yeast IP_8_ dynamics *in vivo*. 1,5-IP_8_ is synthesized *in vivo* from 5-IP_7_ by the N-terminal kinase domain of the bifunctional kinase-pyrophosphatase Asp1 (3, 4). *In vitro*, recombinant full-length Asp1 catalyzes futile cycles of 1-phosphate phosphorylation by its kinase component and 1-pyrophosphate hydrolysis by its pyrophosphatase component that result in unproductive consumption of the ATP substrate (5). An H397A mutation in the active site of the C-terminal pyrophosphatase domain of Asp1 restored net 1,5-IP_8_ synthesis by full-length Asp1-H397A to nearly the same specific activity as the isolated Asp1 kinase domain. Inspired by studies of the human ortholog PPIP5K2 (7), Benjamin et al. (5) found that inorganic phosphate, the product of the Asp1 inositol pyrophosphate pyrophosphatase reaction, enables net 1,5-IP_8_ synthesis *in vitro* by full-length wild-type Asp1. Significant activation of 1,5-IP_8_ synthesis was evident at 25 mM phosphate, which is the reported physiological intracellular concentration of orthophosphate in budding yeast grown in phosphate-replete medium (33). Although these findings regarding phosphate’s effect on Asp1 pyrophosphatase *in vitro* provided a simple explanation for how Asp1 might achieve net 1,5-IP_8_ synthesis in the cellular milieu, the present study injects phosphate-sensitive control of SpSiw14 into the mix. To wit, intracellular phosphate levels sufficient to modulate SpSiw14’s inositol pyrophosphatase activities could increase the 5-IP_7_ substrate available to Asp1 and reduce turnover of the 1,5-IP_8_ product.

The present genetic analyses of fission yeast Siw14 are consistent with it playing a role in 1,5-IP_8_ catabolism *in vivo*, insofar as: (i) elimination of Siw14 protein or inactivation of the Siw14 pyrophosphatase by the C189S mutation had no effect *per se* on *S. pombe* growth but was lethal in the absence of the Nudix-type inositol pyrophosphate pyrophosphatase enzyme Aps1; and (ii) the synthetic lethality of *siw14*∆ *aps1*∆ depended on the synthesis of 1,5-IP_8_ by the Asp1 kinase. We conclude that SpSiw14 and the Aps1 pyrophosphatases have essential but redundant functions in fission yeast, and that their synthetic lethality is a consequence of the toxic effects of too much 1,5-IP_8_. Copious genetic evidence points to 1,5-IP_8_ action as an agonist of precocious 3′-processing/transcription termination as the basis for 1,5-IP_8_ toxicosis (21 – 24). We surmise that this is also the case for *siw14*∆ *aps1*∆ synthetic lethality, which was consistently suppressed by loss-of-function mutations of components of the fission yeast 3′-processing/termination machinery. RNA analyses and monitoring of *PHO* gene expression indicate that the *aps1*∆ deletion leads to increased expression of *pho1* by relieving flanking lncRNA-mediated transcription interference with the *pho1* promoter via precocious lncRNA termination. By contrast, the *siw14*∆ deletion does not elicit such a phenotype on its own. An outstanding challenge is to pinpoint the gene(s) dysregulated by excess 1,5-IP_8_ in *siw14*∆ *aps1*∆ and other lethal IP_8_ pyrophosphatase mutants (21, 22) that are responsible for toxicity.

## MATERIALS AND METHODS

### Recombinant *S. pombe* Siw14

The ORF encoding full-length Siw14 was PCR amplified from *S. pombe* cDNA with primers that introduced a BamHI site immediately flanking the start codon and a XhoI site downstream of the stop codon. A truncated ORF encoding Siw14-(79-287) was generated by PCR amplification with a sense-strand primer that introduced a BamHI site overlying the codon for Ser79. The PCR products were digested and inserted between the BamHI and XhoI sites of pET28b-His_10_Smt3 to generate T7 RNA polymerase-based expression plasmids encoding the Siw14-(1-287) and Siw14-(79-287) polypeptides fused to an N-terminal His_10_Smt3 tag. A missense Cys189Ser mutation was introduced into the expression plasmids by two-stage overlap extension PCR with mutagenic primers. All plasmid inserts were sequenced to verify the fusion junctions and exclude the presence of unwanted mutations.

Wild-type and mutant pET28b-His_10_Smt3-Siw14 plasmids were transfected into *E. coli* BL21(DE3) cells. Cultures (1 L) amplified from single kanamycin-resistant transformants were grown at 37°C in Terrific Broth containing 50 µg/mL kanamycin until the *A*_600_ reached 0.72–0.78. The cultures were chilled on ice for 1 h, adjusted to 2.2% (vol/vol) ethanol and 0.5 mM isopropyl-β-D-thiogalactopyranoside, and then incubated for 16 h at 17°C with constant shaking. The cells were harvested by centrifugation and stored at −80°C. All subsequent steps were performed at 4°C. Thawed cells were resuspended in 25 mL of buffer L (50 mM Tris-HCl, pH 7.5, 500 mM NaCl, 25 mM imidazole, 10% glycerol) and half a tablet of cOmplete EDTA-free Protease Inhibitor Cocktail (Roche). The cells were lysed by sonication, and the insoluble material was removed by centrifugation at 38,000 × *g* for 30 min. The supernatant was mixed for 1 h with 3 mL of nickel-nitrilotriacetic acid (Ni-NTA) agarose resin (Qiagen) that had been equilibrated with buffer L. The resin was recovered by centrifugation and washed twice with 30 mL of buffer L. The resin was centrifuged again, resuspended in 15 mL of buffer L, and poured into a column. After washing the column with 15 mL of buffer L, the bound material was eluted with 6 mL of buffer L containing 300 mM imidazole. The polypeptide compositions of the flow-through and eluate fractions were monitored by SDS-PAGE. The 300 mM imidazole eluate fractions containing His_10_-Smt3-Siw14 were supplemented with Smt3-specific protease Ulp1 [Ulp1/His_10-_Smt3-Siw14 ratio of 1:425 (wt/wt)] and then dialyzed overnight against 2 L of buffer D (50 mM Tris-HCl, pH 7.5, 250 mM NaCl, 25 mM imidazole, 5% glycerol). The dialysates were mixed for 1 h with 3 mL of Ni-NTA agarose resin that had been equilibrated with buffer D. Tag-free Siw14 proteins were recovered in the flow-through fractions. Protein concentration was determined from the *A*_280_ measured with a Nanodrop spectrophotometer (Thermo Scientific), applying a molar extinction coefficient of 24,910 M^−1^/cm for full-length Siw14 and 24,660 M^−1^/cm for Siw14-(79-287), as calculated by Protparam. The yields at this purification step of Siw14-(1-287), Siw14-(1-287)-C189S, Siw14-(79-287), and Siw14-(79-287)-C189S were 12, 20, 16, and 34 mg per liter of bacterial culture, respectively. The tag-free Siw14 preparations were concentrated by centrifugal ultrafiltration (Amicon Ultra-15; 10 kDa cutoff) to 8–14 mg/mL (in a 2-mL volume) and then further purified by gel-filtration through a 125-mL 16/60 HiLoad Superdex 200 column (GE Healthcare) equilibrated in buffer containing 20 mM Tris-HCl, pH 7.5, 100 mM NaCl, and 2 mM dithiothreitol (DTT) at a flow rate of 0.5 mL/min while collecting 2 mL fractions. The peak Siw14 fractions were pooled and concentrated by centrifugal ultrafiltration (Amicon Ultra-15; 10 kDa cutoff) to 2.5–10 mg/mL. Protein concentration was determined from the *A*_280_ measured with a Nanodrop spectrophotometer, applying molar extinction coefficients as described above.

### *p*-Nitrophenylphosphatase activity

Reaction mixtures (50 µL) containing 25 mM Tris-acetate, pH 5.0, 1 mM DTT, 10 mM (500 nmol) *p*-nitrophenylphosphate, and Siw14 protein as specified in the figure legends were incubated at 37°C. The reactions were quenched by adding 0.95 mL of 1 M Na_2_CO_3_. Release of *p*-nitrophenol was determined by measuring *A*_410_ and interpolating the value to a *p*-nitrophenol standard curve.

### Inorganic pyrophosphatase activity

Reaction mixtures (100 µL) containing 25 mM Tris-acetate, pH 5.0, 1 mM DTT, 2 mM (200 nmol) sodium pyrophosphate, and Siw14 protein as specified in the figure legends were incubated at 37°C. The reactions were quenched by adding 1 mL of Malachite Green Reagent (Enzo Life Sciences), followed by a 20-min incubation at room temperature. Phosphate release was determined by measuring *A*_620_ and interpolating the value to a phosphate standard curve.

### Inorganic polyphosphatase activity

Reaction mixtures (10 µL) containing 50 mM Tris-acetate, pH 5.0, 0.2 mM poly-P_45_ (Sigma, Cat # S4379-500MG, Lot # SLBX2788), and Siw14 protein as specified in the figure legends were incubated for 30 min at 37 °C. Reactions were terminated by mixing with an equal volume of 2× Orange G loading buffer (10 mM Tris-HCl, pH 7.0, 1 mM EDTA, 30% glycerol, 0.05% Orange G). The products were analyzed by electrophoresis at 4°C through a 20-cm 36% polyacrylamide gel containing 80 mM Tris-borate (pH 8.3) and 1 mM EDTA for 2.5 h at 10 W constant power. The gel was washed briefly with water and then stained with a solution of 0.1% toluidine blue (Sigma), 20% methanol, and 0.2% glycerol, followed by destaining in 20% methanol.

Alternatively, reaction mixtures (20 µL) containing 50 mM Tris-acetate, pH 5.0, 0.2 mM poly-P_45_ (Sigma, Cat # S4379-500MG, Lot # SLCM4102), and Siw14 protein as specified in the figure legends were incubated for 30 min at 37°C. Reactions were quenched by adding 1 mL of Malachite Green Reagent (Enzo Life Sciences), followed by a 20-min incubation at room temperature. Phosphate release was determined by measuring *A*_620_ and interpolating the value to a phosphate standard curve. The total phosphate content of the poly-P_45_ substrate was measured after digestion for 30 min at 37°C with calf intestine alkaline phosphatase (40 U; NEB).

### Inositol pyrophosphate pyrophosphatase activity

Reaction mixtures containing 30 mM Tris-acetate, pH 5.0, 25 or 50 mM NaCl, 0.25 or 0.5 mM inositol pyrophosphate (1-IP_7_, 5-IP_7_, or 1,5-IP_8_), 0 or 1 mM MgCl_2_, and Siw14 protein as specified in the figure legends were incubated for 30 min at 37°C. The reactions were terminated by adding an equal volume of 2× Orange G loading buffer. The products were analyzed by electrophoresis at 4°C through a 20-cm 36% polyacrylamide gel containing 80 mM Tris-borate (pH 8.3) and 1 mM EDTA for 3 h at 10 W constant power. The inositol polyphosphates were visualized by staining the gel with toluidine blue, as described above.

### Allelic replacement at the *siw14* locus

We constructed strains harboring marked *siw14-WT* and *siw14-C189S* alleles. First, we generated a pKS-based plasmid carrying a *siw14* integration cassette marked with *hygMX*. The cassette consisted of the following elements, proceeding from 5′ to 3′: (i) a 619-bp segment of genomic DNA 5′ of the *siw14^+^* start codon; (ii) a 1107-bp segment encompassing the *siw14* ORF and introns; (iii) a 269-bp segment harboring the *nmt1* ^+^ transcription termination signal; (iv) a *hygMX* gene conferring resistance to hygromycin; and (v) a 748-bp segment of genomic DNA 3′ of the *siw14* ^+^ stop codon. The integration cassette for *siw14-C189S* was generated by replacing a restriction fragment spanning the Cys189 codon in the wild-type integration cassette with a restriction fragment containing the *C189S* missense mutation. The *siw14* ORFs were sequenced to exclude the presence of unwanted mutations. The integration cassettes (WT and C189S) were excised from plasmids and transfected into haploid *S. pombe* cells. Hygromycin-resistant transformants were selected and analyzed by Southern blotting to verify marker integration at the *siw14* locus. The *siw14* ORFs in the *siw14-WT-hygMX* and *siw14-C189S-hygMX* strains were amplified by PCR and sequenced to confirm the *siw14* genotypes.

### Tests of mutational synergies

*siw14*∆ haploids were mixed on malt agar with haploids of the opposite mating type bearing differentially marked mutations in genes involved in inositol pyrophosphate metabolism (*asp1*, *aps1*) and inositol pyrophosphate sensing (*spx1*), RNA 3′-processing and Pol2 transcription termination (*ctf1*, *dis2*, *ppn1*, *swd22*, *ssu72*, *rhn1*), CTD prolyl isomerization (*pin1*), or pan-heptad mutations in the Pol2 CTD (*T4A*) to allow mating and sporulation. After affirming the presence of asci by microscopy and ensuing treatment with glusulase (which breaks down the ascus wall to release spores and kills any residual unmated vegetative cells), the spores were counted in a hemocytometer and then subjected to random spore analysis (34). Spores (~1,000) were plated in parallel on YES agar and on medium selective for the marked mutant alleles, and the plates were incubated at 30°C. A large number of viable drug-resistant progeny were screened by replica-plating for the presence of the second drug resistance marker gene or by sequentially replica-plating from YES to different drug-selective media. Wild-type (unmarked) and differentially marked single mutant alleles were recovered at the expected frequencies. A finding that no haploids with both marker genes were recovered after 6 to 8 days of incubation at 30°C was taken to indicate synthetic lethality. Growth phenotypes of viable double-mutant strains were assessed in parallel with the individual single mutants and wild-type cells at different temperatures (20°C to 37°C). Fission yeast cultures were grown in YES liquid medium at 30°C until *A*_600_ reached 0.6–0.9. The cultures were adjusted to a final *A*_600_ of 0.1, and 3 µL aliquots of serial fivefold dilutions were spotted on YES agar. The plates were photographed after incubation for 2 d at 34°C, 2.5 days at 30°C and 37°C, 4 days at 25°C, and 6 days at 20°C. A list of the fission yeast strains employed in this study is compiled in Table S4.

### Transcriptome profiling by RNA-seq

RNA was isolated from *S. pombe* wild-type and *siw14*∆ cells that were grown in liquid YES medium at 30°C to an *A*_600_ of 0.5 to 0.6. Cells were harvested by centrifugation, and total RNA was extracted via the hot phenol method. The integrity of total RNA was gauged with an Agilent Technologies 2100 Bioanalyzer. The Illumina TruSeq stranded mRNA sample preparation kit was used to purify poly(A)^+^ RNA from 500 ng of total RNA and to carry out the subsequent steps of poly(A)^+^ RNA fragmentation, strand-specific cDNA synthesis, indexing, and amplification. Indexed libraries were normalized and pooled for paired-end sequencing performed using a NOVASeq 6000 system. FASTQ files bearing paired-end reads of length 51 bases were mapped to the *S. pombe* genome (ASM294v2.28) using HISAT2-2.1.0 with default parameters (35). The resulting SAM files were converted to BAM files using Samtools (36). Count files for individual replicates were generated with HTSeq-0.10.0 (37) using exon annotations from Pombase (GFF annotations, genome-version ASM294v2; source “ensembl”). RPKM analysis and pairwise correlations (Tables S1 and S2) were performed as described previously (38). Differential gene expression and fold change analysis were performed in DESeq2 (39). The cutoff for further evaluation was set for genes that had an adjusted *P*-value (Benjamini-Hochberg corrected) of ≤0.05 and were up or down by at least twofold in *siw14*∆ versus wild type. Genes were further filtered on the following criteria: (i) genes that were ≥2-fold up and the average normalized read count for the *siw14*∆ strain was ≥100 and (ii) genes that were ≥2-fold down and the average normalized read count for the wild-type strain was ≥100.

## Data Availability

The RNA-seq data in this publication have been deposited in NCBI’s Gene Expression Omnibus and are accessible through GEO Series accession number GSE232247.

## SUPPLEMENTAL MATERIAL

Supplemental figures and tables mbio.02056-23-s0001.pdf Tables S1, S2, S3, and S4. Figures S1 and S2.

ASM does not own the copyrights to Supplemental Material that may be linked to, or accessed through, an article. The authors have granted ASM a non-exclusive, world-wide license to publish the Supplemental Material files. Please contact the corresponding author directly for reuse.
