# Supplementary material for: Activities, substrate specificity, and genetic interactions of fission yeast Siw14, a cysteinyl-phosphatase-type inositol pyrophosphatase
Source: mBio. 2023 Sep 29;14(5):e02056-23. doi: 10.1128/mbio.02056-23 (PMC10653929; doi:10.1128/mbio.02056-23)
Supplement: Supplemental figures and tables — Tables S1, S2, S3, and S4. Figures S1 and S2. [file mbio.02056-23-s0001.pdf]

Supplemental Material

Activities, substrate specificity, and genetic interactions of fission yeast Siw14, a cysteinyl-phosphatase-type inositol pyrophosphatase

Ana M. Sanchez, Beate Schwer, Nikolaus Jork, Henning Jessen, and Stewart Shuman

Supplemental Tables S1, S2, S3, S4

Supplemental Figures S1, S2

Sample	Total Paired Reads	Mapped Reads
WT (1)	20,262,658	17,933,794 (88%)
WT (2)	19,335,273	17,068,516 (88%)
WT (3)	18,961,556	16,612,814 (88%)
*siw14*Δ (1)	21,010,644	18,008,068 (86%)
*siw14*Δ (2)	21,310,189	18,574,597 (87%)
*siw14*Δ (3)	19,708,969	16,990,972 (86%)

Table S1. RNA-seq read counts for triplicate biological replicates.

Sample pairs	Pearson Coefficient
WT (1) vs (2)	0.968
WT (2) vs (3)	0.974
WT (1) vs (3)	0.975
*siw14*Δ (1) vs (2)	0.981
*siw14*Δ (2) vs (3)	0.984
*siw14*Δ (1) vs (3)	0.982

Table S2. RNA-seq data reproducibility between biological replicates.

Table S3. Transcriptome profiling of *siw14*Δ cells by poly(A)[+] RNA-seq.

Gene ID	Gene Name	log2 fold change (*siw14*Δ/*WT*)	Adjusted p value
SPBC14F5.01		1.13994	0.007258
SPACUNK4.14	*mdb1*	1.02159	0.000257
SPBC17A3.03c	*siw14*	-7.65550	4.57E-211
SPAC27D7.03c	*mei2*	-1.64694	0.001697
SPAC15E1.02c		-1.40318	0.005297
SPAP8A3.04c	*hsp9*	-1.36203	0.001697
SPAC637.03		-1.24609	0.014389
SPACUNK4.17		-1.13839	0.010978
SPBC1683.09c	*frp1*	-1.13410	0.000982
SPCC70.08c		-1.06210	0.014683
SPBC725.10	*tps0*	-1.03359	0.007028
SPBC16E9.16c	*lsd90*	-1.00726	0.039732
SPAC26H5.09c		-1.00226	0.002264
SPNCRNA.130	*omt3* lncRNA	-2.77207	0.001622
SPNCRNA.747	*osh3*-antisense-1	-1.34473	0.001877
SPNCRNA.803	*hrp1*-antisense-1	-1.44841	0.002604
SPNCRNA.872	*eta2*-antisense-1	-1.05613	0.002727
SPNCRNA.888	*end4*-antisense-1	-1.00139	0.012388

List of annotated 2 protein-coding RNAs that were up-regulated and 11 protein-coding RNAs that were down-regulated by at least two-fold in *siw14*Δ cells versus wild-type cells, as well as 5 non-coding RNAs (SPNCRNAs) that were downregulated at least two-fold.

Table S4. *S. pombe* strains used in this study.

Strain	Genotype	Source
AS478	*h- Sp1 leu1-32 ura4-D18 his3-D1 ade6-m216*	Sanchez et al. 2019 (21)
AS479	*h+ Sp2 leu1-32 ura4-D18 his3-D1 ade6-m210*	Sanchez et al. 2019 (21)
AS2302	*h- siw14Δ::hygMX*	Sanchez et al. 2023 (25)
AS2303	*h+ siw14Δ::hygMX*	Sanchez et al. 2023 (25)
AS2549	*h- siw14Δ::kanMX*	Sanchez et al. 2023 (25)
AS2550	*h- siw14Δ::natMX*	Sanchez et al. 2023 (25)
AS1884	*h- aps1Δ::kanMX*	Sanchez et al. 2019 (21)
AS2185	*h+ asp1-H397A::natMX*	Sanchez et al. 2019 (21)
AS2152	*h- aps1Δ::natMX ctf1Δ::kanMX*	Sanchez et al. 2019 (21)
AS2153	*h- aps1Δ::natMX rhn1Δ::kanMX*	Sanchez et al. 2019 (21)
AS2157	*h- aps1Δ::natMX ppn1Δ::hygMX*	Sanchez et al. 2019 (21)
AS2562	*h+ aps1Δ::hygMX pin1Δ::natMX*	Sanchez et al. 2020 (28)
AS2212	*h- aps1Δ::natMX asp1-D333A::kanMX*	Sanchez et al. 2019 (21)
AS2155	*h- aps1Δ::natMX dis2Δ::ura4+*	Sanchez et al. 2019 (21)
AS2158	*h+ aps1Δ::natMX swd22Δ::hygMX*	Sanchez et al. 2019 (21)
AS2154	*h+ aps1Δ::natMX ssu72-C13S::kanMX*	Sanchez et al. 2019 (21)
AS2162	*h- aps1Δ::kanMX rpb1-T4A::natMX*	Sanchez et al. 2019 (21)
BS401	*h+ aps1Δ::hygMX spx1Δ::kanMX*	Schwer et al. 2022 (24)
ASY187	*h+ siw14-WT::hygMX*	This study
ASY188	*h- siw14-WT::hygMX*	This study
ASY189	*h+ siw14-C189S::hygMX*	This study
ASY190	*h- siw14-C189S::hygMX*	This study
ASY197	*h- siw14Δ::kanMX*	This study
AS2312	*h- siw14Δ::hygMX asp1-H397A::natMX*	This study
ASY199	*h+ siw14Δ::hygMX aps1Δ::natMX ctf1Δ::kanMX*	This study
ASY203	*h+ siw14Δ::hygMX aps1Δ::natMX rhn1Δ::kanMX*	This study
ASY202	*h+ siw14Δ::kanMX aps1Δ::natMX ppn1Δ::hygMX*	This study
ASY201	*h- siw14Δ::kanMX aps1Δ::hygMX pin1Δ::natMX*	This study
ASY200	*h- siw14Δ::hygMX aps1Δ::natMX asp1-D333A::kanMX*	This study
ASY198	*h+ siw14Δ::hygMX aps1Δ::natMX dis2Δ::ura4+*	This study
BS823	*h+ siw14Δ::kanMX aps1Δ::natMX swd22Δ::hygMX*	This study
BS825	*h- siw14Δ::hygMX aps1Δ::natMX ssu72-C13S::kanMX*	This study
BS824	*h- siw14Δ::hygMX aps1Δ::kanMX rpb1-T4A::natMX*	This study
BS828	*h- siw14Δ::natMX aps1Δ::hygMX spx1Δ::kanMX*	This study

All strains are *leu1-32 ura4-D18 his3-D1* and either *ade6-m216 or ade6-m210*.

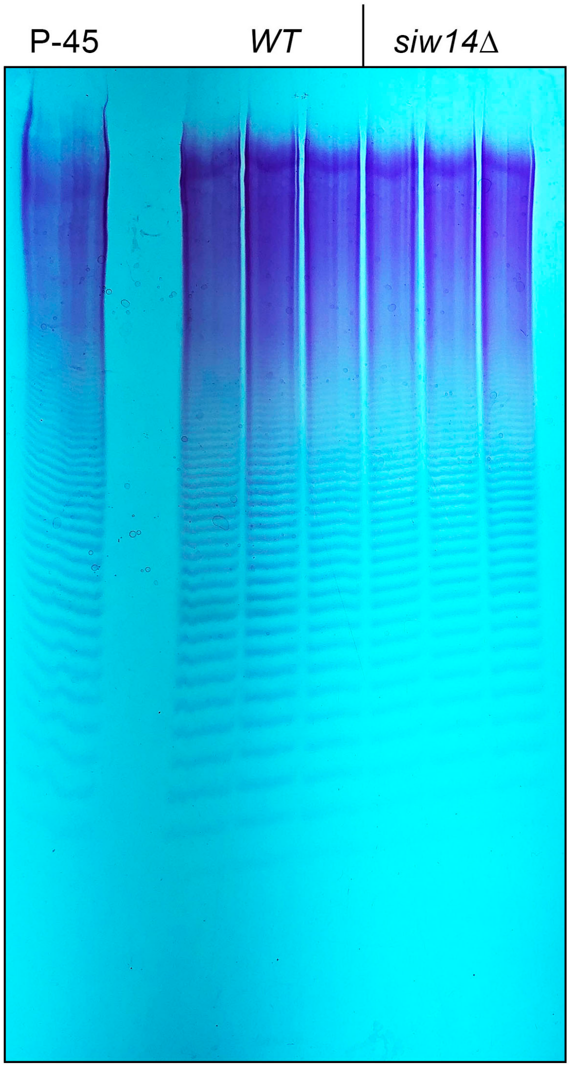

Figure S1. Polyphosphate analysis of wild-type and *siw14*Δ cells. *S. pombe* wild-type and *siw14*Δ cells were grown in YES medium at 30°C. Aliquots (5 A_{600} units) of logarithmically growing triplicate cultures of each strain were harvested by centrifugation, washed with cold water and stored at -80°C. The cell pellets were resuspended in 400 µl AE buffer (50 mM sodium acetate pH 5.2, 10 mM EDTA) and added to 300 µl phenol (equilibrated in 10 mM Tris-HCl, pH 8.0) plus 40 µl of 10% (w/v) SDS. The samples were mixed vigorously and incubated at 65°C for 5 min and then placed on ice. Chloroform (300 µl) was added, and the mixed samples were centrifuged for 5 min at room temperature at 17,000g. The aqueous phase (~450 µl) was collected, extracted with phenol/chloroform and chloroform, and then ethanol precipitated at -20°C overnight. The precipitates were washed with 70% ethanol, dried and resuspended in 75 µl TE (10 mM Tris-HCl, pH 7.0, 1 mM EDTA; 15 µl per 1 A_{600} unit of cells). Aliquots (10 µl) were supplemented with 3x Orange G dye (10 mM Tris-HCl, pH 7.0, 1 mM EDTA, 30% glycerol, 0.1% [w/v] Orange G) and then analyzed by electrophoresis through a 36% polyacrylamide gel in TBE (80 mM Tris-borate, 1 mM EDTA) at 4°C for 17 h at 5 mA. The gel was stained with toluidine blue (0.05% [w/v] toluidine blue in 20% methanol, 2% glycerol) and photographed after de-staining. Lane P-45 contains commercial polyphosphate (average chain length 45; Sigma S4379).

```
          . ..  .    ....  .   .. ..     ..... .  . . . .. .     .  .
spo       PDNFGVVYPGIIYRSACPRASNFNFLE-SLHIRTIISLRQEEYSEEDLHYFTKHHINYYH   141
sce       PENFSHV-VGEIYRSSFPRQENFSFLHERLKLKSILVLIPEEYPQENLNFLKLTGIKLYQ   177
ath       PLNFSMV-DNGIFRSGFPDSANFSFLQ-TLGLRSIIYLCPEPYPESNLQFLKSNGIRLFQ   113

            . . .            . .      .   . ....  . ...... ....... ...
spo       IAMPGSKHRKNDCISSSSNPDISDVDDLVRKTLQLLLNKENWPVLLHCSRGKHRTGIVIG   201
sce       VGMSGNKE-----------PFVNIPSHLLTKALEIVLNPANQPILIHCNRGKHRTGCLIG   226
ath       FGIEGNKE-----------PFVNIPDHKIRMALKVLLDEKNHPVLIHCKRGKHRTGCLVG   162

          ... . .  .     ..  .   . .  .. ..  .
spo       CLRALMNWPVGNRLQEYISFSHPKEREVDEEYIQNFSSDPSLKSSL--NDLKRYISDSSS   259
sce       CIRKLQNWSLTMIFDEYRRFAFPKARALDQQFIEMYDDDEIKRIASKNNWLPLQW*    281
ath       CLRKLQKWCLTSIFDEYQRFAAAKARVSDQRFMEIFDVSSFSHIPMSFSCSIR*      215

spo       ELADVVLSSESPTVQAATVNETCRSPGS  287
```

Figure S2. Primary structure similarity between *S. pombe* (spo), *S. cerevisiae* (sce), and *A. thaliana* (ath) Siw14 proteins. The amino acid sequences of the C-terminal cysteinyl-phosphatase domains of the three Siw14 proteins were aligned in Clustal. 71 positions of side chain identity/similarity are indicated by dots above the alignment. The cysteine nucleophile and the arginine that coordinates the scissile phosphate are highlighted in yellow. Conserved residues that, in the plant enzyme, coordinate the non-scissile phosphates of 5-IP_7 (pdb 7MOE) are highlighted in green.
